# Feasibility evaluation of tumor treating fields for brainstem gliomas

**DOI:** 10.1007/s11060-026-05697-y

**Published:** 2026-07-06

**Authors:** Anthony Yulin Chen, Ryan Shah, William Shi, Noa Urman, Nadav Shapira, Nicholas Avgeropoulos, Patrick Conlon, Neil Mookerjee, Wenyin Shi

**Affiliations:** 1 Sidney Kimmel Medical College, Thomas Jefferson University, Philadelphia, PA USA; 2 Emory University, Atlanta, GA USA; 3 Novocure Ltd, Haifa, Israel; 4 Novocure Inc, Portsmouth, NH UK; 5 Department of Radiation Oncology, Thomas Jefferson University, 111 S 11 ST, Suite G301, Philadelphia, PA 19017 USA

**Keywords:** Tumor treating fields, Brainstem, Glioma, Treatment planning, Dosimetry

## Abstract

**Purpose:**

Tumor treating fields (TTFields) are FDA-approved for supratentorial glioblastoma, but feasibility in infratentorial tumors remains poorly defined. This simulation study evaluated TTFields dose in brainstem gliomas using patient-specific modeling with scalp-only transducer arrays.

**Methods:**

MRI and CT imaging from seven patients with brainstem gliomas were used for TTFields planning with MAXPOINT^®^ (Novocure, Switzerland). Clinical target volume (CTV) was defined as enhancing tumor on T1 post-contrast MRI (Gross tumor volume, GTV) plus a 3 mm peritumoral expansion. The platform optimized scalp-only array layouts, and finite element calculations generated maps of local minimum field intensity (LMiFI, V/cm) and local minimum power density (LMiPD, mW/cm³). Values were compared against a standard, unplanned layout using one-sided paired t-tests, with LMiFI ≥ 1.0 V/cm as a therapeutic reference.

**Results:**

MAXPOINT-optimized layouts achieved significantly higher LMiFI than the standard layout across all regions (all *p* ≤ 0.019). GTV median LMiFI was 1.1 vs. 1.0 V/cm (*p* = 0.019). CTV median LMiFI was 1.1 vs. 1.0 V/cm (*p* = 0.002). Brainstem median LMiFI was 1.3 vs. 1.1 V/cm (*p* = 0.002). Posterior fossa showed the largest difference: median LMiFI 1.5 vs. 1.2 V/cm (*p* < 0.001). All optimized LMiFI values met or exceeded the therapeutic reference. LMiPD was also significantly higher with MAXPOINT^®^ across all regions (all *p* ≤ 0.006).

**Conclusion:**

This simulation study demonstrates that patient-specific modeling can achieve favorable TTFields intensities in brainstem gliomas using scalp-only arrays, supporting prospective clinical evaluation for infratentorial tumors.

**Supplementary Information:**

The online version contains supplementary material available at 10.1007/s11060-026-05697-y.

## Introduction

Tumor Treating Fields (TTFields) are alternating electric fields that disrupt mitosis and inhibit tumor growth through dielectrophoretic effects and microtubule interference [1, 2]. The landmark EF-14 trial established TTFields as standard therapy for newly diagnosed supratentorial glioblastoma (GBM), demonstrating significant improvements in both progression-free survival (median 6.7 vs. 4.0 months) and overall survival (median 20.9 vs. 16.0 months) when added to maintenance temozolomide following standard chemoradiotherapy [3]. These findings, together with results from prior trials such as EF-11 [4], led to FDA approval and NCCN guidelines recommendation of TTFields for both newly diagnosed and recurrent supratentorial GBM.

Given these benefits in supratentorial GBM, extending TTFields to other glioma locations is of interest. Brainstem gliomas represent a particularly compelling target given the limited treatment options and poor outcomes in this population [5]. These tumors present unique therapeutic challenges because their deep midline location and close proximity to critical cranial nerve nuclei and long white matter tracts often preclude safe surgical resection, while concerns regarding toxicity to eloquent brainstem structures limit the ability to escalate radiation dose [5–7]. The literature for TTFields treatment of infratentorial gliomas is limited. Lok et al. evaluated a cerebellar GBM using finite element analysis, while Ramirez-Fort et al. modeled two brainstem gliomas using generic prototype head models [8, 9]. Neither study reported power density, a dosimetric parameter quantifying the rate of energy delivered to the tissue, which, along with field intensity, has been shown to associate with survival from secondary analyses of the EF-14 trial [2, 10, 11]. These studies also proposed nonconventional neck-extended arrays that have not been tested for clinical implementation. Segar et al. achieved high field coverage using intracranial electrodes, but this requires surgical implantation [12]. Together, these studies suggest theoretical feasibility, but do not establish whether therapeutic doses can be achieved in brainstem gliomas using practical, scalp-only arrays.

To rigorously address this question, we utilized the FDA-approved MAXPOINT^®^ system (Novocure, Switzerland) rather than traditional NovoTAL-based planning. NovoTAL (Novocure, LLC) selects array configurations based on MRI-derived morphometric measurements and a library of precomputed field distributions, without incorporating patient-specific anatomy or displaying the resulting field distribution [13]. It is also only available for supratentorial disease and cannot perform planning for brainstem and posterior fossa lesions. MAXPOINT^®^, by contrast, generates patient-specific finite element models from individualized MRI and CT segmentation, enabling three-dimensional calculation and visualization of electric field intensity (V/cm) and power density (mW/cm^3^) distributions. It also allows for direct comparison of multiple array configurations per patient using quantitative dose-volume metrics, supporting individualized plan selection. Unlike NovoTAL, MAXPOINT^®^ can perform planning for infratentorial tumors, including those of the brainstem and posterior fossa.

Using this platform, we evaluated the dosimetric performance of MAXPOINT-generated array layouts for patients with brainstem gliomas as compared with a standard array layout, and whether local minimum field intensity (LMiFI) for the patient-specific layouts exceeded a reference value of 1 V/cm, approximating a range for therapeutic activity. We characterized metrics of LMiFI and local minimum power density (LMiPD) within the target volume, brainstem, and posterior fossa.

## Methods

### Patients

This study was performed in accordance with the ethical standards of the institutional and/or national research committee and with the 1964 Helsinki Declaration and its later amendments or comparable ethical standards. All protocols were approved by the Institutional Review Board of Thomas Jefferson University (IRB# 18D.480). The requirement for informed consent was waived by the Institutional Review Board due to the retrospective nature of the study.

Seven consecutive patients with brainstem gliomas who received radiation treatment from 2022 to 2024 at Thomas Jefferson University meeting eligibility criteria were included. Inclusion criteria required radiographic diagnosis of brainstem glioma and availability of thin-cut volumetric MRI and CT imaging suitable for segmentation-based planning. There were no additional exclusion criteria.

### TTFields planning software

The MAXPOINT^®^ TTFields planning system (Novocure, Switzerland) was used for all simulations. INE TTFields arrays were modeled for treatment planning.

### Target segmentation

Thin-cut volumetric T1 post-contrast and FLAIR MRI sequences, along with CT imaging of the head, were imported into the MAXPOINT^®^ planning platform and fused for segmentation. Gross tumor volume (GTV) was defined as contrast-enhancing tumor on T1 post-contrast MRI, including necrotic tumor and any associated surgical cavity. The peritumoral brain zone (PBZ) was defined as a 3 mm uniform expansion of the GTV, consistent with manufacturer guidelines for volume definition and prior TTFields dosimetric studies [11]. The clinical target volume (CTV) was defined as the union of the GTV and PBZ. We note that these GTV, PBZ, and CTV definitions follow TTFields-planning convention and differ from radiotherapy target definitions, where the CTV typically encompasses FLAIR-defined disease.

Additional regions of interest, including the brainstem and posterior fossa, were contoured to assess TTFields dose distribution within critical infratentorial anatomy. As this was a simulation study, no patient received TTFields treatment.

### Array layout generation and dose mapping

Segmentation was manually performed as described above. Automated whole-brain finite element modeling within MAXPOINT^®^ generated voxel-level tissue models, including gray matter, white matter, skull, scalp, and cerebrospinal fluid, and assigned appropriate electric conductivity values for treatment planning. The automated tissue model and the segmentation of brain structures were reviewed and approved for anatomical accuracy by an experienced radiation oncologist prior to dose calculation.

TTFields treatment planning was performed within MAXPOINT^®^, which computes patient-specific electric field and power density distributions based on segmented anatomy. The software optimized transducer array layouts by iteratively adjusting placement to maximize LMiPD to the CTV while maintaining realistic scalp positioning. Five distinct treatment plans with different array layouts, plus one supplementary plan, were generated for each patient.

For each plan, three-dimensional electric field (V/cm) and power density (mW/cm³) maps were generated. LMiFI and LMiPD were recorded for segmented targets and regions of interest, including the GTV, PBZ, CTV, brainstem, and posterior fossa. Cohort values are reported as medians with ranges.

Dose-volume histograms and field and power density distributions were reviewed to compare the five treatment plans and supplementary plan. The two plans demonstrating the highest target intensity and optimal coverage were designated as the primary and alternative plans by the reviewing physician.

For reference, a standard array layout was also evaluated for comparison. This configuration represents the conventional generic array placement strategy and does not incorporate patient-specific MRI anatomy or individualized field optimization. It is automatically generated and consists of symmetrically placed left-right and anterior-posterior array pairs, adapted to each patient’s overall head size. Three-dimensional electric field (V/cm) and power density (mW/cm³) maps were also generated. LMiFI and LMiPD were recorded for segmented targets and regions of interest, including the GTV, PBZ, CTV, brainstem, and posterior fossa.

### Statistical analysis

This was a simulation study evaluating TTFields dose distribution within treatment targets and regions of interest in patients with brainstem gliomas. Dosimetric results were summarized using medians with ranges. LMiFI and LMiPD values for MAXPOINT-optimized layouts were compared against values from a standard, unplanned array layout using one-sided paired t-tests, based on the directional hypothesis that patient-specific optimization would yield higher field intensities and power densities than a generic layout. Statistical significance was defined as *p* < 0.05. Additionally, achieved LMiFI values were compared against a reference value of 1.0 V/cm, based on historical measures of field intensity associated with accelerated therapeutic effects across multiple tumor types [2, 10, 11].

## Results

### TTFields planning feasibility

**Fig. 1:**
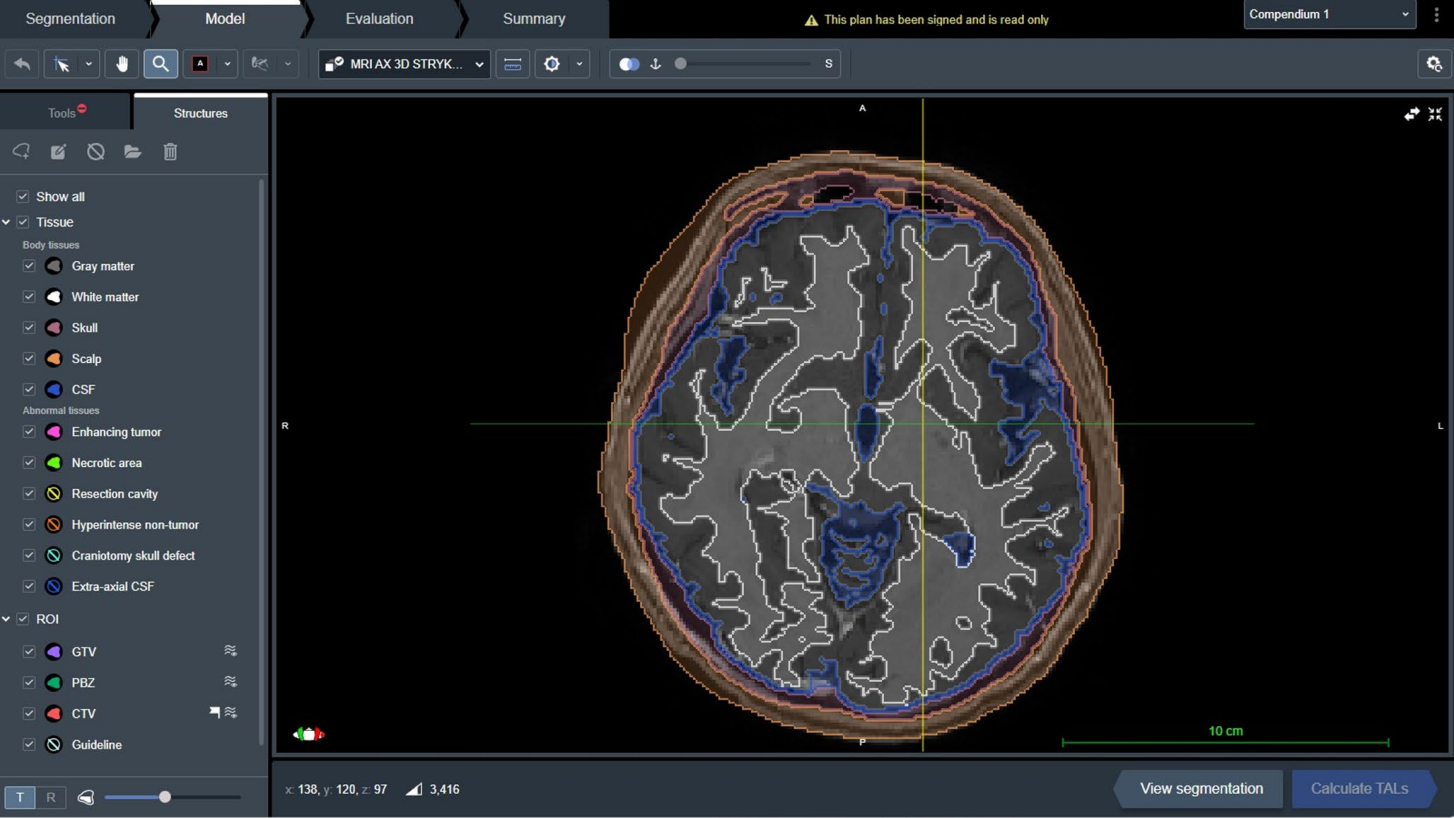
Representative tissue model of a sample case. Patient-specific segmentation of gray matter, white matter, cerebrospinal fluid (CSF), and skull was performed automatically to generate the anatomical model used for treatment planning and electric field simulations

Segmentation-based TTFields treatment planning using the MAXPOINT^®^ system was done for seven patients with brainstem gliomas (see Supplementary Information S1 for tumor and patient characteristics). Automated tissue modeling generated consistent voxel-level delineation of gray matter, white matter, cerebrospinal fluid, skull, and scalp (Fig. 1). Following tissue model approval, MAXPOINT^®^ optimized scalp-only transducer array layouts for each patient and performed finite element dose calculations.

**Fig. 2:**
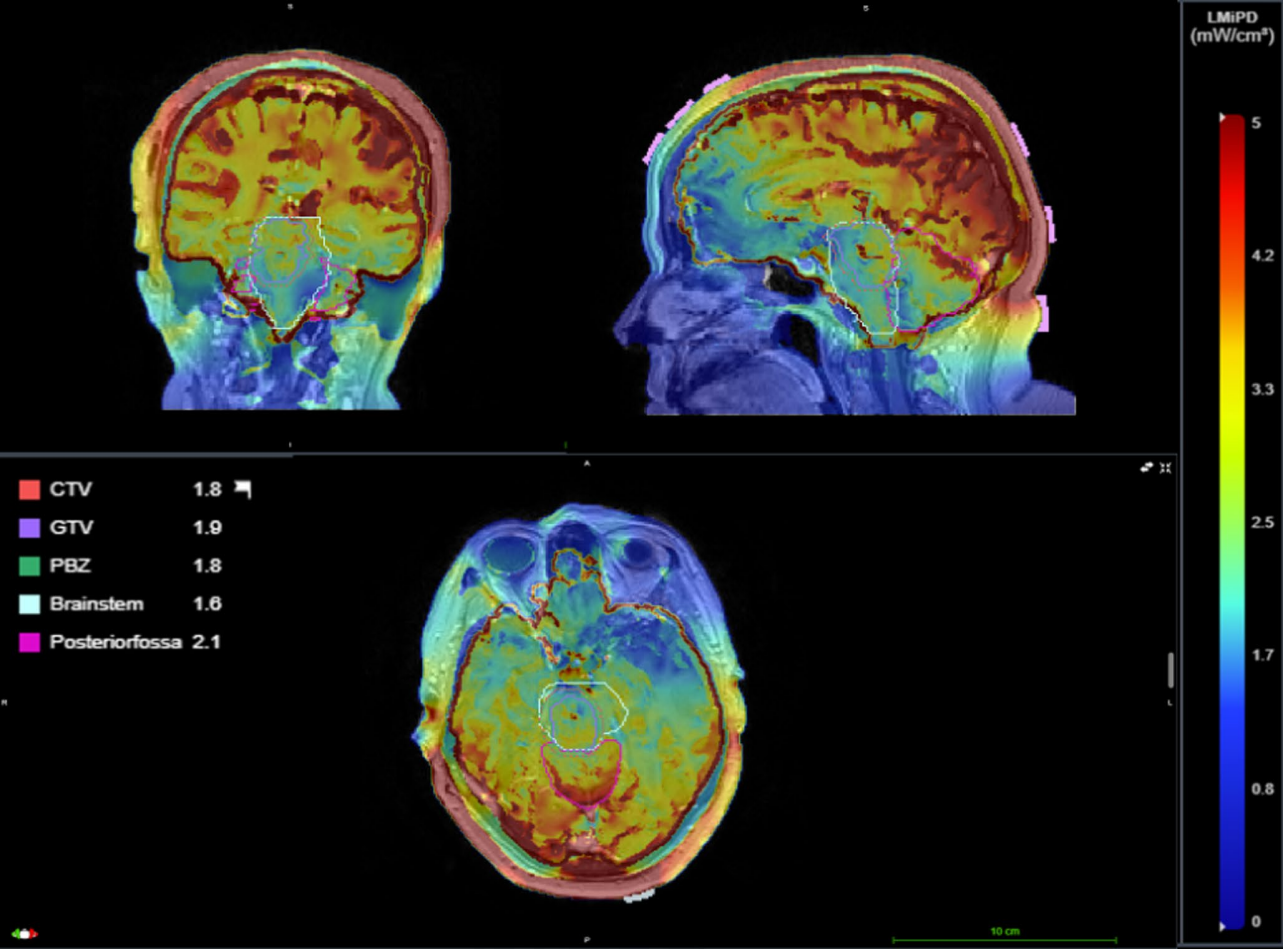
Electric field and power density distributions from parieto-occipital array configurations. Field penetration into the GTV, CTV, and surrounding brainstem structures is visualized across axial, sagittal, and coronal planes

**Fig. 3:**
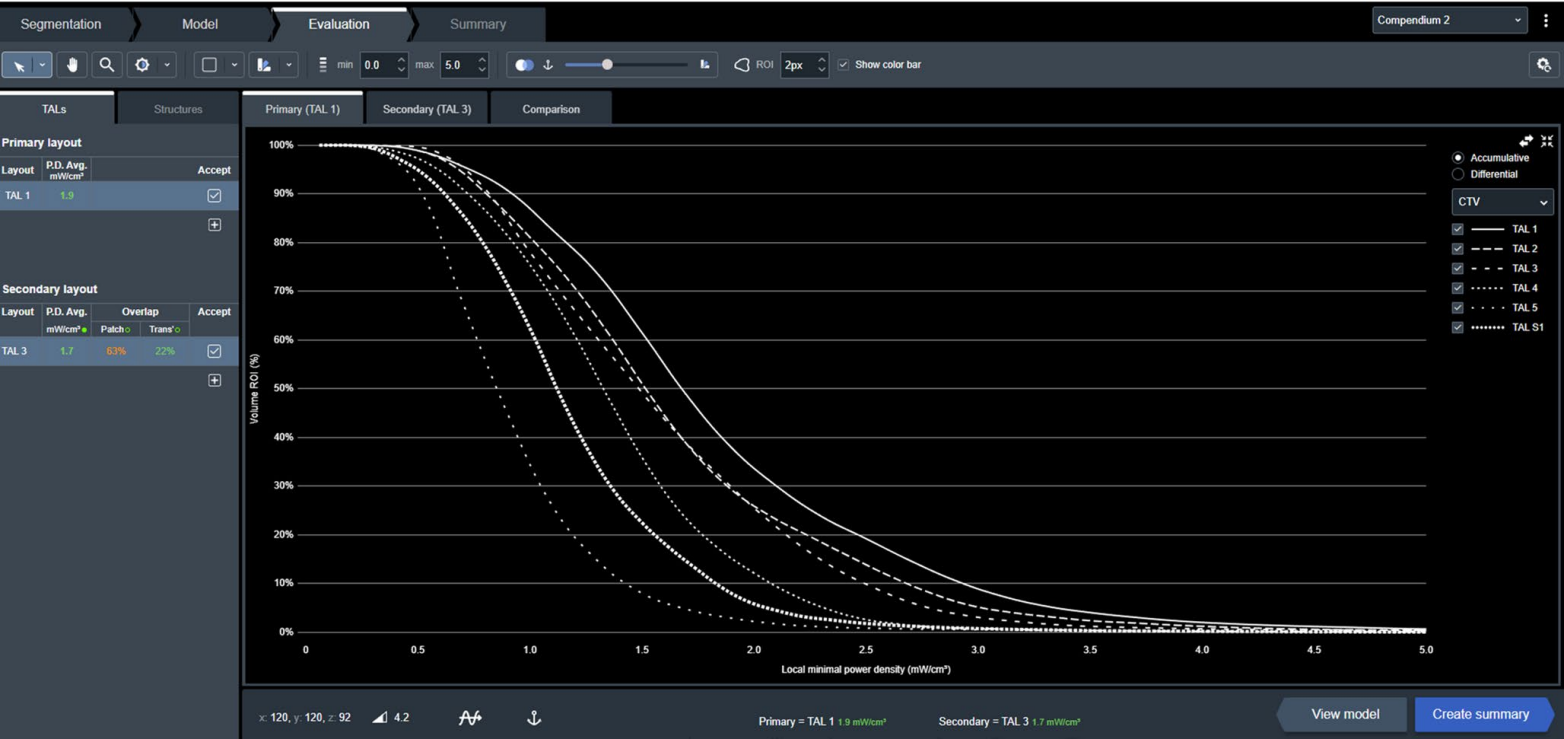
Sample case cumulative dose–volume histogram (DVH) comparing five MAXPOINT^®^-generated plans and one supplemental plan. The figure illustrates dosimetric differences among candidate array layouts, enabling comparison of target coverage and selection of the optimal treatment configuration

**Fig. 4:**
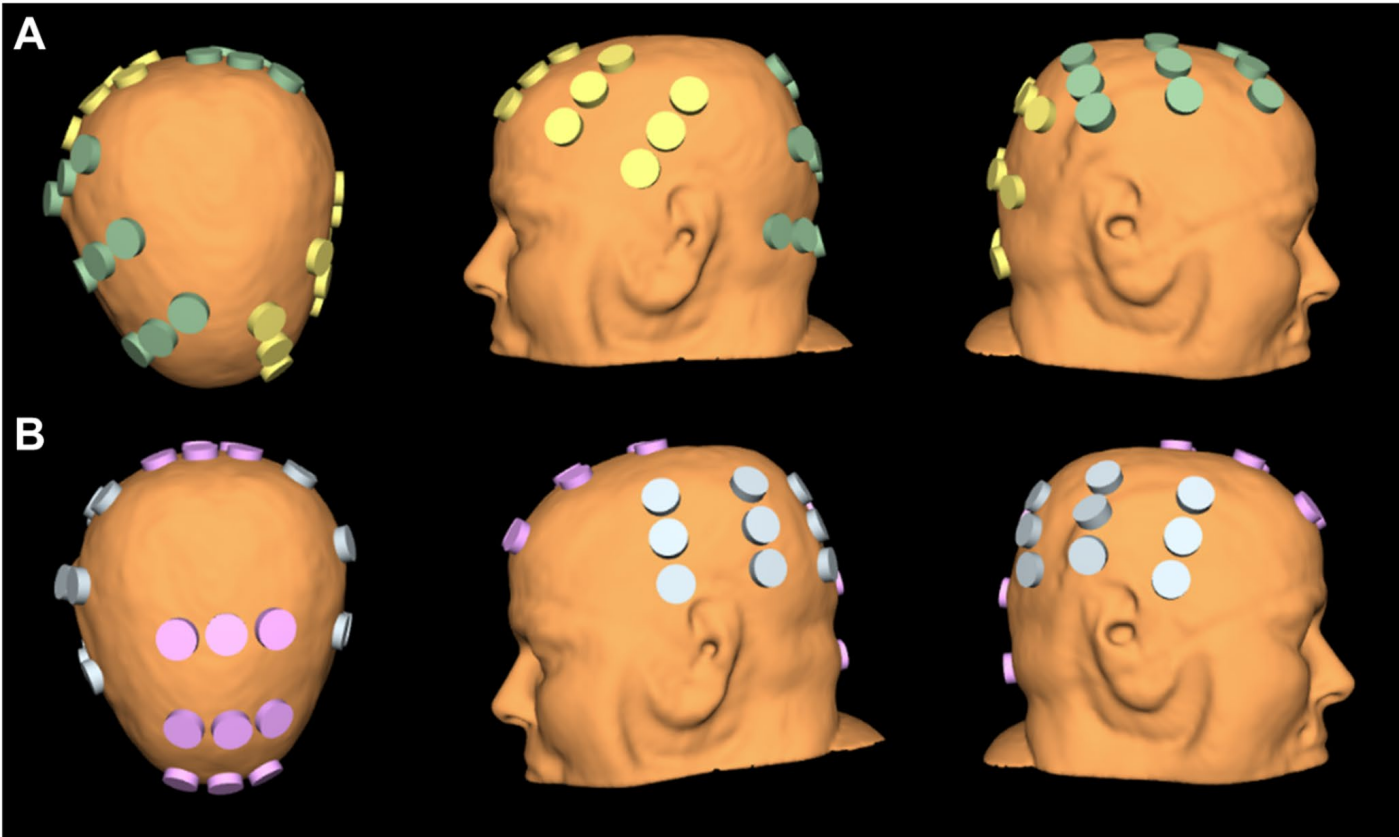
Representative array layout generated for a treatment plan (**A**), and for the unplanned, generic layout (**B**). The image illustrates the positioning and orientation of the transducer arrays on a representative patient’s scalp as determined by the planning software

Five distinct treatment plans, plus one supplementary plan, were generated for each patient. Three-dimensional maps of electric field intensity and power density maps (Fig. 2), dose-volume histograms (Fig. 3), and array layout visualizations (Fig. 4) were available to guide plan evaluation and selection. The plan with the highest LMiFI and LMiPD was selected as the approved plan and used for TTFields dosimetry evaluation (see Supplementary Information S2 for detailed dosimetric comparisons between plans).

### TTFields dose to targets and regions of interest

Across seven patients, therapeutically favorable TTFields distributions were consistently achieved, with electric field intensity metrics meeting or exceeding predefined reference values (LMiFI ≥ 1.0 V/cm) across all target volumes, including the GTV, PBZ, and CTV. MAXPOINT-optimized layouts achieved significantly higher LMiFI and LMiPD compared to the standard layout across all target volumes and regions of interest (all *p* ≤ 0.019; Table 1). Within the CTV, the median LMiFI was 1.1 vs. 1.0 V/cm (*p* = 0.002) and the median LMiPD was 1.4 vs. 1.2 mW/cm³ (*p* = 0.006) for MAXPOINT-optimized versus standard layouts, respectively, indicating adequate field penetration and energy deposition in the target volume (Table 1).

**Table 1:** TTFields dosimetry summary. Median dose values are reported for MAXPOINT-optimized and standard array layout configurations

Region	LMiPD (mW/cm^3^), median (range)			LMiFI (V/cm), median (range)			
	MAXPOINT	Standard	*P*-value^†^		MAXPOINT	Standard	*P*-value^†^
GTV	1.4 (1.0–1.9)	1.2 (0.7–1.6)	0.003		1.1 (1.0–1.5)	1.0 (0.9–1.4)	0.019
PBZ	1.4 (1.0-1.9)	1.3 (0.8–1.6)	0.004		1.1 (1.1–1.4)	1.0 (1–1.5)	0.008
CTV	1.4 (1.0–1.8)	1.2 (0.8–1.6)	0.006		1.1 (1.1–1.5)	1.0 (0.9–1.4)	0.002
Brainstem	1.4 (1.2–1.6)	1.1 (0.9–1.4)	0.001		1.3 (1.1–1.5)	1.1 (1–1.4)	0.002
Posterior Fossa	2.1 (1.9–2.4)	1.3 (1–1.7)	< 0.001		1.5 (1.4–1.6)	1.2 (1–1.3)	< 0.001

† P-values from one-sided, paired t-test

CTV: clinical target volume; GTV: gross tumor volume; LMiFI: local minimum field intensity; LMiPD: local minimum power density; PBZ: peritumoral brain zone

Comparable dosimetric profiles were observed in the brainstem, where MAXPOINT-optimized layouts achieved a median LMiFI of 1.3 vs. 1.1 V/cm (*p* = 0.002) and median LMiPD of 1.4 vs. 1.1 mW/cm³ (*p* = 0.001), demonstrating effective coverage of this anatomically complex and clinically critical region. The largest differences were observed in the posterior fossa, with median LMiFI of 1.5 vs. 1.2 V/cm (*p* < 0.001) and LMiPD of 2.1 vs. 1.3 mW/cm³ (*p* < 0.001) for MAXPOINT-optimized versus standard layouts, respectively. These findings suggest that TTFields can achieve robust and potentially therapeutic field distributions even in deep-seated and posterior intracranial regions, and that patient-specific optimization provides a statistically significant dosimetric advantage over standard array placement (Table 1).

## Discussion

We demonstrate that favorable therapeutic TTFields dose can be achieved in brainstem gliomas and posterior fossa using non-invasive, scalp-only array configurations compatible with current clinical practice. MAXPOINT-optimized plans achieved statistically significantly higher TTFields dose to the CTV compared to the standard layout, with average LMiFI exceeding historical values associated with therapeutic activity for gliomas [2, 10, 11]. These findings support exploration of TTFields for infratentoiral gliomas, a site for which it is not currently approved, as NCCN guidelines restrict TTFields use to supratentorial GBM [14].

Our findings contribute significantly to the small pool of literature on TTFields feasibility for gliomas in brainstem and posterior fossa. Previous studies have been limited by smaller sample sizes and reliance on unconventional array configurations. Lok et al. demonstrated that posterior displacement of arrays could increase cerebellar field coverage by 46.6%, but this was validated in only one patient and required placements extending to the neck [8]. Ramirez-Fort et al. proposed similar neck-based configurations for two brainstem gliomas, demonstrating intensities above 1 V/cm in both vertical and horizontal field directions in 95% of the infratentorium [9]. While effective computationally, such layouts are not patient-specific; thus, dose distribution and array configurations may not be optimal for the patient [15, 16]. This is particularly important because TTFields survival benefit increases with duration of use, requiring patients to wear the device continuously for ≥ 18 h per day with minimal interruptions [16, 17]. Real-world adherence data demonstrate that achieving this target remains challenging, with median usage rates of 65.5% reported in clinical practice, well below the intended 75% threshold [18]. This adherence gap further supports the use of practical, scalp-only configurations that minimize patient burden, though practical considerations specific to posterior fossa array placement warrant consideration, such as scalp preparation and potential discomfort during supine sleep. Segar et al. circumvented these issues by modeling intracranial electrode placement, achieving high field intensities but requiring surgical implantation with its associated procedural risks [12]. Our findings suggest that neither unconventional transcranial placements nor invasive approaches are necessary for TTFields treatment of brainstem gliomas.

The consistent achievement of survival-associated dosimetric levels across seven diverse brainstem tumors suggests that TTFields delivery to infratentorial regions may be feasible without device modifications or novel array designs. This is significant as a validated patient-specific modeling framework for non-conventional array layouts is still lacking, making it difficult to assess whether such configurations can reliably achieve therapeutic doses. Importantly, this modeling was only possible due to MAXPOINT^®^’s ability to perform patient-specific brainstem and posterior fossa tumor planning, which is a key distinction to the NovoTAL system that is limited to supratentorial planning. Given that TTFields have demonstrated tolerability and quality of life preservation with no systemic toxicity, clinical validation of these dosimetric findings could establish TTFields as a non-invasive option for brainstem gliomas [19, 20].

From a safety perspective, the higher values observed in superficial supratentorial regions (Fig. 2) reflect proximity to the scalp arrays and mirror the field distribution routinely delivered to supratentorial glioblastoma patients during standard TTFields therapy. As TTFields selectively disrupt mitosis in dividing cells while largely sparing post-mitotic neurons [2, 10], exposure of supratentorial tissue to these intensities has not been associated with central nervous system toxicity in the EF-14 trial or in large post-marketing safety surveillance. Most reported adverse effects have been dermatologic [3, 16, 20].

This study has several limitations that should be addressed. First, although computational modeling demonstrates feasibility, it does not guarantee clinical efficacy. Achieving therapeutic field intensities is necessary but not sufficient for tumor response, which depends on numerous cellular and molecular factors not captured by simulation. Second, the dosimetric reference value used (LMiFI ≥ 1.0 V/cm) was derived from preclinical studies and may not directly translate to brainstem gliomas [11, 21, 22]. Third, no biologically or clinically meaningful threshold has been established for LMiPD in brainstem gliomas. So, while LMiPD differences were statistically significant, their clinical significance cannot be determined from dosimetric data alone. Fourth, this dosimetric data was calculated with a 3 mm PBZ consistent with similar prior studies and manufacturer’s instructions, though larger peritumoral margins applied in clinical practice may not achieve equivalent TTFields intensities. Fifth, the use of one-sided paired t-tests, while appropriate for the directional hypothesis, and the absence of correction for multiple comparisons should be considered when interpreting the reported p-values. Sixth, our models assume idealized tissue conductivity, which can be affected by treatment-related changes and peritumoral edema [23]. Lastly, although this is the largest infratentorial modeling study to date, seven cases remain a modest sample size. Of note, our modeling was performed using the array hardware available prior to November 2024, when the FDA approved lighter, thinner, and more flexible arrays designed to improve patient comfort. Given unchanged array geometry and placement, we expect similar dosimetric findings with the updated hardware.

This study provides a platform for future clinical modeling and prospective clinical investigation. Early-phase trials assessing safety, tolerability, and preliminary efficacy of TTFields in brainstem glioma patients would be a logical next step. The recent expansion of TTFields to sites like metastatic non-small cell lung cancer and brain metastases demonstrates its increasing applicability beyond the original supratentorial targets, supporting continued investigation in infratentorial tumors [24, 25]. Although our cohort only included adult patients, a similar dosimetric rationale may extend to pediatric diffuse midline glioma, including H3 K27-altered diffuse intrinsic pontine glioma (DIPG), for which effective therapies remain limited [7]. Early clinical experience indicates that scalp-array TTFields is safe and feasible in children with high-grade glioma, supporting future investigation of non-invasive infratentorial delivery in this population [26].

## Conclusion

This study demonstrates that favorable therapeutic TTFields doses can be achieved in brainstem gliomas and posterior fossa tumors using clinically practical, scalp-only array configurations. These feasibility findings support expanding clinical investigation of TTFields beyond current supratentorial indications. Prospective trials evaluating safety and efficacy in patients with brainstem gliomas and posterior fossa tumors are warranted.

## Supplementary Information

Below is the link to the electronic supplementary material.

Supplementary Material 1

## Data Availability

No datasets were generated or analysed during the current study.

## References

[1] Giladi M, Schneiderman RS, Porat Y, Munster M, Itzhaki A, Mordechovich D, Cahal S, Kirson ED, Weinberg U, Palti Y (2014) Mitotic disruption and reduced clonogenicity of pancreatic cancer cells in vitro and in vivo by tumor treating fields. Pancreatology 14:54–63. 10.1016/j.pan.2013.11.009 24555979 10.1016/j.pan.2013.11.009

[2] Kirson ED, Gurvich Z, Schneiderman R, Dekel E, Itzhaki A, Wasserman Y, Schatzberger R, Palti Y (2004) Disruption of cancer cell replication by alternating electric fields. Cancer Res 64:3288–3295 15126372 10.1158/0008-5472.can-04-0083

[3] Stupp R, Taillibert S, Kanner A, Read W, Steinberg D, Lhermitte B, Toms S, Idbaih A, Ahluwalia MS, Fink K, Di Meco F, Lieberman F, Zhu JJ, Stragliotto G, Tran D, Brem S, Hottinger A, Kirson ED, Lavy-Shahaf G, Weinberg U, Kim CY, Paek SH, Nicholas G, Bruna J, Hirte H, Weller M, Palti Y, Hegi ME, Ram Z (2017) Effect of Tumor-Treating Fields Plus Maintenance Temozolomide vs Maintenance Temozolomide Alone on Survival in Patients With Glioblastoma: A Randomized Clinical Trial. JAMA 318:2306–2316. 10.1001/jama.2017.18718 29260225 10.1001/jama.2017.18718 PMC5820703

[4] Stupp R, Wong ET, Kanner AA, Steinberg D, Engelhard H, Heidecke V, Kirson ED, Taillibert S, Liebermann F, Dbaly V, Ram Z, Villano JL, Rainov N, Weinberg U, Schiff D, Kunschner L, Raizer J, Honnorat J, Sloan A, Malkin M, Landolfi JC, Payer F, Mehdorn M, Weil RJ, Pannullo SC, Westphal M, Smrcka M, Chin L, Kostron H, Hofer S, Bruce J, Cosgrove R, Paleologous N, Palti Y, Gutin PH (2012) NovoTTF-100A versus physician’s choice chemotherapy in recurrent glioblastoma: a randomised phase III trial of a novel treatment modality. Eur J Cancer 48:2192–2202. 10.1016/j.ejca.2012.04.011 22608262 10.1016/j.ejca.2012.04.011

[5] Wummer B, Woodworth D, Flores C (2021) Brain stem gliomas and current landscape. J Neurooncol 151:21–28. 10.1007/s11060-020-03655-w 33398531 10.1007/s11060-020-03655-w

[6] Guillamo JS, Monjour A, Taillandier L, Devaux B, Varlet P, Haie-Meder C, Defer GL, Maison P, Mazeron JJ, Cornu P, Delattre JY (2001) Association des Neuro-Oncologues d’Expression F Brainstem gliomas in adults: prognostic factors and classification. Brain 124: 2528–2539 10.1093/brain/124.12.2528 10.1093/brain/124.12.2528 11701605

[7] Lo Greco MC, Marano G, La Rocca M, Acquaviva G, Milazzotto R, Liardo RLE, Basile A, Foti PV, Palmucci S, David E, Parisi S, Pontoriero A, Pergolizzi S, Spatola C (2025) Latest Advancements in the Management of H3K27M-Mutant Diffuse Intrinsic Pontine Glioma: A Narrative Review. Cancers (Basel) 17. 10.3390/cancers17030420 10.3390/cancers17030420 PMC11815860 39941789

[8] Lok E, San P, Liang O, White V, Wong ET (2020) Finite element analysis of Tumor Treating Fields in a patient with posterior fossa glioblastoma. J Neurooncol 147:125–133. 10.1007/s11060-020-03406-x 31989489 10.1007/s11060-020-03406-x PMC7076058

[9] Ramirez-Fort MK, Naveh A, McClelland S 3rd, Gilman CK, Fort M, Mendez M, Matta J, Bomzon Z, Lange CS (2021) Computational simulations establish a novel transducer array placement arrangement that extends delivery of therapeutic TTFields to the infratentorium of patients with brainstem gliomas. Rep Pract Oncol Radiother 26:1045–1050. 10.5603/RPOR.a2021.0132 10.5603/RPOR.a2021.0132 PMC8726444 34992879

[10] Kirson ED, Dbaly V, Tovarys F, Vymazal J, Soustiel JF, Itzhaki A, Mordechovich D, Steinberg-Shapira S, Gurvich Z, Schneiderman R, Wasserman Y, Salzberg M, Ryffel B, Goldsher D, Dekel E, Palti Y (2007) Alternating electric fields arrest cell proliferation in animal tumor models and human brain tumors. Proc Natl Acad Sci U S A 104:10152–10157. 10.1073/pnas.0702916104 17551011 10.1073/pnas.0702916104 PMC1886002

[11] Ballo MT, Urman N, Lavy-Shahaf G, Grewal J, Bomzon Z, Toms S (2019) Correlation of Tumor Treating Fields Dosimetry to Survival Outcomes in Newly Diagnosed Glioblastoma: A Large-Scale Numerical Simulation-Based Analysis of Data from the Phase 3 EF-14 Randomized Trial. Int J Radiat Oncol Biol Phys 104:1106–1113. 10.1016/j.ijrobp.2019.04.008 31026557 10.1016/j.ijrobp.2019.04.008

[12] Segar DJ, Bernstock JD, Arnaout O, Bi WL, Friedman GK, Langer R, Traverso G, Rampersad SM (2023) Modeling of intracranial tumor treating fields for the treatment of complex high-grade gliomas. Sci Rep 13:1636. 10.1038/s41598-023-28769-9 36717682 10.1038/s41598-023-28769-9 PMC9886948

[13] Mikic N, Gentilal N, Cao F, Lok E, Wong ET, Ballo M, Glas M, Miranda PC, Thielscher A, Korshoej AR (2024) Tumor-treating fields dosimetry in glioblastoma: Insights into treatment planning, optimization, and dose-response relationships. Neurooncol Adv 6:vdae032. 10.1093/noajnl/vdae032 38560348 10.1093/noajnl/vdae032 PMC10981464

[14] Nabors LB, Hattangadi-Gluth J, Horbinski C, Portnow J (2025) NCCN CNS Tumor Guidelines Update for 2024. Neuro Oncol 27:595–596. 10.1093/neuonc/noae267 39693230 10.1093/neuonc/noae267 PMC11889715

[15] Onken J, Goerling U, Heinrich M, Pleissner S, Krex D, Vajkoczy P, Misch M (2019) Patient Reported Outcome (PRO) Among High-Grade Glioma Patients Receiving TTFields Treatment: A Two Center Observational Study. Front Neurol 10:1026. 10.3389/fneur.2019.01026 31681134 10.3389/fneur.2019.01026 PMC6797850

[16] Lacouture ME, Anadkat MJ, Ballo MT, Iwamoto F, Jeyapalan SA, La Rocca RV, Schwartz M, Serventi JN, Glas M (2020) Prevention and Management of Dermatologic Adverse Events Associated With Tumor Treating Fields in Patients With Glioblastoma. Front Oncol 10:1045. 10.3389/fonc.2020.01045 32850308 10.3389/fonc.2020.01045 PMC7399624

[17] Toms SA, Kim CY, Nicholas G, Ram Z (2019) Increased compliance with tumor treating fields therapy is prognostic for improved survival in the treatment of glioblastoma: a subgroup analysis of the EF-14 phase III trial. J Neurooncol 141:467–473. 10.1007/s11060-018-03057-z 30506499 10.1007/s11060-018-03057-z PMC6342854

[18] Jelgersma C, Alsolivany J, Akkas G, Wasilewski D, Gastl B, Misch M, Capper D, Kaul D, Bullinger L, Vajkoczy P, Onken J (2024) Real-world experience with TTFields in glioma patients with emphasis on therapy usage. Front Oncol 14:1430793. 10.3389/fonc.2024.1430793 39839796 10.3389/fonc.2024.1430793 PMC11747310

[19] Taphoorn MJB, Dirven L, Kanner AA, Lavy-Shahaf G, Weinberg U, Taillibert S, Toms SA, Honnorat J, Chen TC, Sroubek J, David C, Idbaih A, Easaw JC, Kim CY, Bruna J, Hottinger AF, Kew Y, Roth P, Desai R, Villano JL, Kirson ED, Ram Z, Stupp R (2018) Influence of Treatment With Tumor-Treating Fields on Health-Related Quality of Life of Patients With Newly Diagnosed Glioblastoma: A Secondary Analysis of a Randomized Clinical Trial. JAMA Oncol 4:495–504. 10.1001/jamaoncol.2017.5082 29392280 10.1001/jamaoncol.2017.5082 PMC5885193

[20] Mrugala MM, Shi W, Iwomoto F, Lukas RV, Palmer JD, Suh JH, Glas M (2024) Global post marketing safety surveillance of Tumor Treating Fields (TTFields) therapy in over 25,000 patients with CNS malignancies treated between 2011–2022. J Neurooncol 169:25–38. 10.1007/s11060-024-04682-721 10.1007/s11060-024-04682-7 PMC11269345 38949692

[21] Reyes-Botero G, Giry M, Mokhtari K, Labussiere M, Idbaih A, Delattre JY, Laigle-Donadey F, Sanson M (2014) Molecular analysis of diffuse intrinsic brainstem gliomas in adults. J Neurooncol 116:405–411. 10.1007/s11060-013-1312-2 24242757 10.1007/s11060-013-1312-2

[22] Chen LH, Pan C, Diplas BH, Xu C, Hansen LJ, Wu Y, Chen X, Geng Y, Sun T, Sun Y, Zhang P, Wu Z, Zhang J, Li D, Zhang Y, Wu W, Wang Y, Li G, Yang J, Wang X, Xu C, Wang S, Waitkus MS, He Y, McLendon RE, Ashley DM, Yan H, Zhang L (2020) The integrated genomic and epigenomic landscape of brainstem glioma. Nat Commun 11:3077. 10.1038/s41467-020-16682-y 32555164 10.1038/s41467-020-16682-y PMC7299931

[23] Lang ST, Gan LS, McLennan C, Monchi O, Kelly JJP (2020) Impact of Peritumoral Edema During Tumor Treatment Field Therapy: A Computational Modelling Study. IEEE Trans Biomed Eng 67:3327–3338. 10.1109/TBME.2020.2983653 32286953 10.1109/TBME.2020.2983653

[24] Leal T, Kotecha R, Ramlau R, Zhang L, Milanowski J, Cobo M, Roubec J, Petruzelka L, Havel L, Kalmadi S, Ward J, Andric Z, Berghmans T, Gerber DE, Kloecker G, Panikkar R, Aerts J, Delmonte A, Pless M, Greil R, Rolfo C, Akerley W, Eaton M, Iqbal M, Langer C, Investigators LS (2023) Tumor Treating Fields therapy with standard systemic therapy versus standard systemic therapy alone in metastatic non-small-cell lung cancer following progression on or after platinum-based therapy (LUNAR): a randomised, open-label, pivotal phase 3 study. Lancet Oncol 24:1002–1017. 10.1016/S1470-2045(23)00344-3 37657460 10.1016/S1470-2045(23)00344-3

[25] Mehta MP, Gondi V, Ahluwalia MS, Roberge D, Sio TTW, Trifiletti DM, Muanza T, Krpan AM, Zhu Z, Ramakrishna NR, Fiveash JB, Metellus P, Yu J, Wang CJ, Jacob J, Freyschlag CF, Csoszi T, Salmaggi A, Taliansky A, Lucas A, Debus J, Brown PD, Harat M (2026) Tumor Treating Fields Therapy After Stereotactic Radiosurgery for Brain Metastases From Non-Small Cell Lung Cancer: Final Results of the Phase 3 METIS Study. Int J Radiat Oncol Biol Phys 124:278–293. 10.1016/j.ijrobp.2025.08.066 41033612 10.1016/j.ijrobp.2025.08.066

[26] Makimoto A, Terashima K, Nishikawa R, Fujisaki H, Kurihara J, Ihara S, Adachi JI, Enokizono M, Mori N, Morikawa Y, Yuza Y (2026) Safety and Efficacy of Tumor-Treating Fields (TTFields) Therapy for Pediatric High-Grade Glioma: Results of a Prespecified Interim Analysis of the First Three Cases. Child (Basel) 13:84. 10.3390/children13010084 10.3390/children13010084 PMC12840331 41597092

