# Supplementary material for: Feasibility evaluation of tumor treating fields for brainstem gliomas
Source: J Neurooncol. 2026 Jul 6;178(3):91. doi: 10.1007/s11060-026-05697-y (PMC13337937; doi:10.1007/s11060-026-05697-y)
Supplement: Supplementary file 1 — Supplementary Material 1 [file 11060_2026_5697_MOESM1_ESM.docx]

**Supplementary Information S1:** Patient and tumor characteristics.

| **Sex** |  |
| --- | --- |
| Male | 2 (28.5%) |
| Female | 5 (71.5%) |
| **Age, (years, median [range])** | 58 (51-67) |
| **Pathology** |  |
| Diffuse Midline Glioma | 6 (85.8%) |
| Astrocytoma, IDHmt, G3 | 1 (14.2%) |
| **Location** |  |
| Pons | 6 (85.8%) |
| Medulla | 1 (14.2%) |
| **Maximum tumor size (cm)** | 1.0-3.9 |
| **Median tumor size (cm)** | 1.9 |
| **GTV (cm^3^, median [range])** | 8.7 (3.8-19.8) |
| **CTV (cm^3^, median [range])** | 18.9 (9.1-33.1) |

CTV: clinical target volume; GTV: gross tumor volume

**Supplementary information S2:** Dose-volume histogram of all plans generated for a representative patient, LiMPD values are displayed for reference.

**
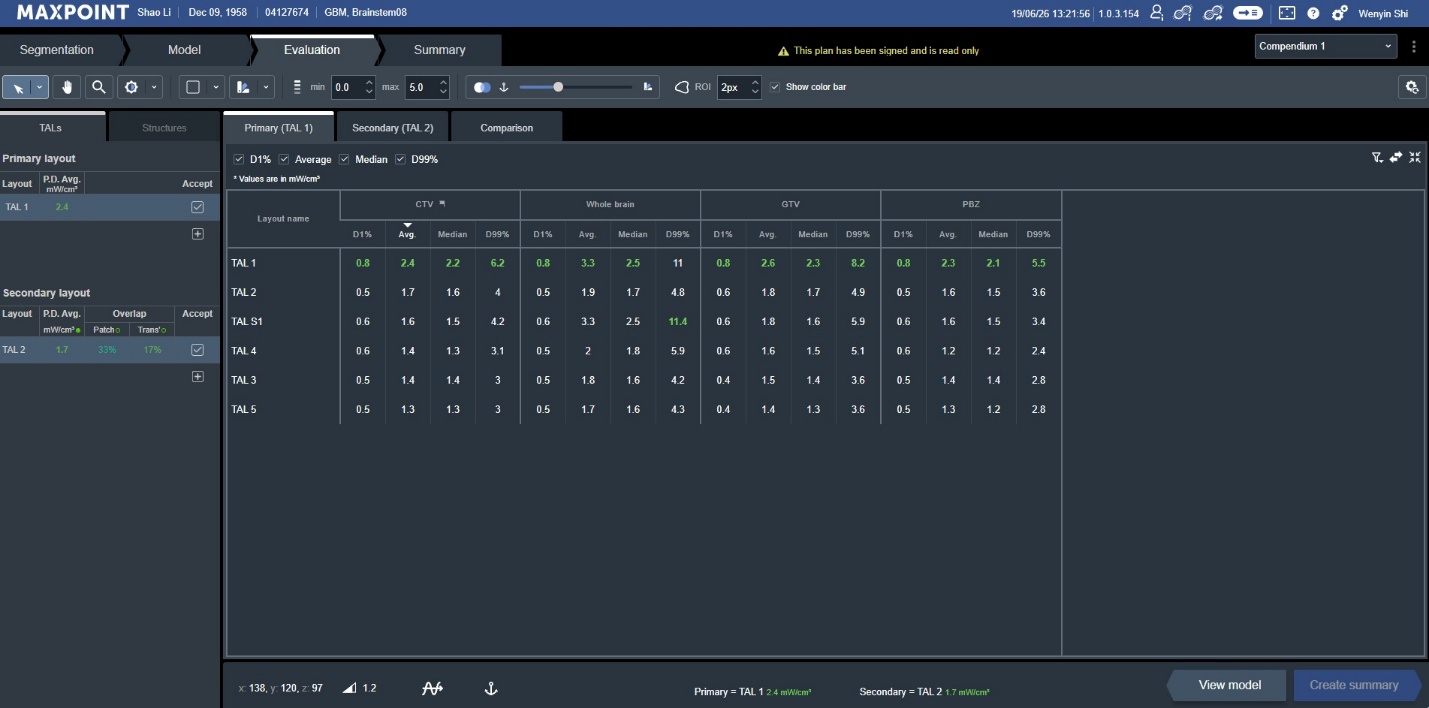
**

CTV: clinical target volume; GTV: gross tumor volume; LMiPD: local minimum power density; PBZ: peritumoral brain zone
